# The Elements of Eco-Connection: A Cross-Cultural Lexical Enquiry

**DOI:** 10.3390/ijerph16245120

**Published:** 2019-12-14

**Authors:** Tim Lomas

**Affiliations:** School of Psychology, University of East London, London E15 4LZ, UK; t.lomas@uel.ac.uk

**Keywords:** eco-connection, nature, language, literacy, cross-cultural

## Abstract

The environment is widely recognised to be in peril, with clear signs of a climate crisis. This situation has many dimensions and factors, but key among them are the often-destructive ways in which humans interact with the natural world. Numerous cultures—particularly more industrialised and/or Western ones—have developed predatory and disconnected modes of interaction. In such modes, nature tends to be constructed as a resource to be exploited (rather than, say, a commonwealth to be protected). However, many people—especially, but not only, in less ‘developed’ nations—have cultivated less destructive modes of relationship. These bonds may be broadly encompassed under the rubric of ‘eco-connection’. In the interests of exploring these latter modes, an enquiry was conducted into adaptive forms of engagement with nature across the world’s cultures. The enquiry focused on untranslatable words, i.e., which lack an exact translation in another language (in this case, English). Through a quasi-systematic search of academic and grey literature, together with additional data collection, over 150 relevant terms were located. An adapted form of grounded theory identified three main dimensions of eco-connection: sacrality, bonding, and appreciation. Such analyses have the potential to promote greater wellbeing literacy with respect to our relationship with nature, both within academia and beyond in the wider culture. This includes enriching the nomological network in psychology, and more broadly building a nature-related vocabulary that is more sustainable and harmonious. In doing so, there may also be benefits to public health, in that developing such literacy could possibly influence people’s engagement with nature itself, leading to more adaptive forms of relationship.

## 1. Introduction

The global environment is increasingly recognised to be in peril, with alarming statistics on the state of the climate emerging almost daily. To give one prominent example, one of the latest reports by the authoritative Intergovernmental Panel on Climate Change [1] charts how glaciers and ice sheets worldwide are already melting rapidly, and argues that the world may only have until 2030 to keep global warming below 1.5 degrees (widely recognised as the threshold past which runaway climate change is likely to occur). Such developments have great existential significance for human beings. As such, the crisis—and our response to it—has a potent psychological dimension, as reflected in emergent concepts such as eco-anxiety [2]. As humans wrestle with this increasingly urgent predicament, various ways of appraising and addressing the situation can be found worldwide, from scientific analyses and technological fixes to moral arguments and public activism. For instance, aligning with the latter, the burgeoning Extinction Rebellion movement focuses on the role of government inaction in fomenting this state of affairs, and aims to compel their action through acts of civil disobedience [3].

Among these varied approaches, a report for the UK government’s Committee on Climate Change argues that one important strategy will be to address how we think about our relationship to nature, psychologically and culturally [4]. Over recent centuries, industrialisation has seen the rise to global prominence and dominance of a mode of relationship that is fundamentally extractive and predatory. In this, nature is constructed as a resource to be exploited (rather than a commonwealth to be protected, for example). This mode of relationship is arguably a key reason why humans have damaged the environment to such an extent that our own survival is threatened, since this way of relating both encourages and justifies such behaviour. Thus, if we are to find more sustainable and adaptive ways of living on this planet, we will need to develop more harmonious and respectful modes of relationship. Crucially, such modes have been cultivated historically, and indeed can still be found, particularly in less-industrialised cultures, but also in industrialised ones (albeit generally in non-hegemonic ways). In that sense, studying and engaging with these modes has the potential to enhance our ‘wellbeing literacy’ [5] with respect to our relationship with nature. This includes helping people find new ways to conceptualise, articulate, rationalise and discuss this relationship. Wellbeing literacy can be broadly defined as “the vocabulary, knowledge and skills that may be intentionally used to maintain or improve the wellbeing of oneself or others” [6]. In that respect, the literacy central to the current paper is one where the ‘others’ in that definition is not restricted to humans, or even sentient beings, but the environment as a whole. Moreover, this literacy in turn may potentially have beneficial public health outcomes by improving the relationship itself.

As such, this paper explores more adaptive and sustainable modes of relationship between humans and the environment, modes which are referred to collectively here under the overarching rubric of ‘eco-connection’. The paper explores these through the innovative device of studying ‘untranslatable’ words. By analysing such words, three main themes pertaining to eco-connection were identified, each with three sub-themes: sacrality (including animism, polytheism and pan(en)theism); bonding (including intertwining, rootedness and longing); and appreciation (including savouring, sensitivity and aesthetics). These themes shall be introduced and explained in depth below. Before that, the paper will elucidate the nature and significance of untranslatable words and outline the method deployed to identify and analyse these. First though, we begin by examining further the idea that recent centuries have been dominated by destructive modes of relationship between humans and nature.

### 1.1. Dominion and Disconnection

There are many possible ways for humans to be in relationship with the natural world, as we shall see below. However, over recent centuries, one particular mode of relationship has become dominant worldwide [7]. This is one characterised by a disconnected, extractive, predatory ethos, where nature is constructed as a resource to be exploited. Before delving into the origins and nature of this mode, a few preparatory remarks are in order.

First, this mode tends to be associated with industrialised nations, and often with Western ones in particular. However, given the complex dynamics of globalisation and cultural change, one can see signs of this mode worldwide, for instance in non-Western industrialised nations. Thus, neither this mode, nor industrialisation, can be characterised as exclusively ‘Western’ phenomena. Second, even if this mode is hegemonic in Western and/or industrialised nations, other modes can still be found within these places (as ‘subcultures’ or subaltern perspectives). Indeed, movements such as Extinction Rebellion are examples of environmental counter-perspectives that have emerged in the West [3]. As such, although some scholars have found value in assigning overarching characteristics to large-scale regions—for instance, Basáñez [8] identifies three ‘hyper-clusters’ of cultures, which focus respectively on honour (mainly African, Islamic, and Christian orthodox cultures), achievement (mainly Asian and Western), and joy (mainly Latin American and Caribbean)—the current study prefers to avoid such broad-brush cultural generalisations where possible. Indeed, many of the words included in the analysis hail from languages of Western and/or industrialised nations. Furthermore, it is precisely this heterogeneity and dynamism within cultures that provides some hope that this dominant mode of relationship can be altered for the better. Finally, it should be emphasised that the destruction of the environment is over-determined, and cannot be traced to single causal factors such as industrialisation or disconnection. For instance, another important factor is over-population, whose damaging effects are observed even in pre-industrial societies, such as Easter Island [9]. Thus, this study does not make any claims for a simple inverse relationship between eco-connection and industrialisation. It is possible that such a relationship does indeed obtain, but this would be an empirical question for future research, and is beyond the scope of this paper to answer.

With all that said, let’s consider this predatory mode of relationship itself. It is also beyond the scope here to exhaustively consider its origins and complexity. However, we can make some relevant points to illustrate the general thesis here. In terms of its roots, many factors arguably contributed to its emergence and dominance. One key influence though is the perspective expressed in the Old Testament (in Genesis 1:26). This is rendered in English (King James Version) as, “And God said, let us make man in our image, after our likeness: and let them have dominion over the fish of the sea, and over the fowl of the air, and over the cattle, and over all the earth, and over every creeping thing that creepeth upon the earth.” Much has been made of this passage by scholars, particularly in relation to the key word dominion, a translation of the Hebrew word radah. Some argue that in Hebrew, while radah can indeed convey a sense of ‘ruling over’, it can also be interpreted beneficently, as a just king may rule wisely over his kingdom [10]. From this latter perspective, the significance with which humans are endowed by God—as alluded to in the passage—confers a duty of care and responsibility towards nature. In that respect, certain movements within Judaism and Christianity have embraced this kind of ‘stewardship’ [11].

Indeed, such stewardship is a mode of eco-connection which has been an important element of many cultures worldwide, including in Western and/or industrialised nations (even if it has not been hegemonic or dominant in such places). This influence is reflected in, and captured by, a significant body of recent literature. For example, showing the presence of stewardship principles in a Western context, Raymond and colleagues interviewed UK farmers, and identified four types of stewardship framings [12]: environmental (farmers conserving or restoring wildlife); primary production (taking care of primary production assets); holistic (farmers as conservationists, primary producers, and managers of landscape values); and instrumental (focusing on financial benefits linked to compliance with agri-environmental schemes). Similarly, a qualitative systematic review by Enqvist et al. uncovered four distinct meanings of stewardship in the literature [13]: ethic, motivation, action and outcome. Mathevet, Bousquet, and Raymond also articulated four main types of stewardship [14]: reformist, adaptive, sustainability and transformative. These are differentiated according to: the role of science; the exploration and integration of the plurality of values; and the capacity to modify values, rules and decision-making system. Evidently then, modes of eco-connection like stewardship can be found both historically and presently, including in Western and/or industrialised contexts.

Historically however, such beneficent perspectives have often been outweighed or overshadowed by less generous interpretations of radah and dominion. These instead focus more on humankind standing apart from nature (rather than being ‘of it’), subjugating it. It is of course a complex picture, given the many traditions and schools of thought in religions such as Judaism and Christianity, and moreover their evolution over time. For instance, Lea argues that, influenced by Judaic and Hellenic traditions, early Christianity upheld a non-exploitative attitude to nature, driven partly by the anti-materialist prescriptions of these influences [15]. However, in the wake of the Renaissance and the Reformation, the dynamic began to shift towards a more extractive relationship. These eras saw the emergence and then dominance of new forms of religious morality, emphasising concerns such as industriousness, instrumental engagement with the material world, and pursuit of personal prosperity and property. Moreover, so central was the Church to the cultural and intellectual climate of Europe at the time, these ideals soon became hegemonic. For instance, Max Weber argued influentially that the ideas, ethics, and practices of Protestantism were key factors in the emergence of capitalism in the West [16]. Relatedly, they helped shaped the burgeoning scientific revolution, which was likewise driven by a systematic focus on the material world [17]. As with capitalism, this was an engagement which tended to view nature as a resourced to be probed, manipulated, or harnessed to the benefit of humankind.

These influences have persisted in the West (and elsewhere), even as the hold of religious traditions themselves has waned. To give some context, in the most recent UK census, 59.3% of the population identified as Christian, a sharp decline from 71.7% in 2001 [18]. Moreover, of those affiliated to the Church, Collings-Mayo and colleagues suggest many see this identification more as a fading ‘inherited cultural memory’ than an ‘active faith’ [19]. However, as scholars such as Jordan Peterson have argued, such ‘cultural memory’ is still very important in shaping who people are [20]. Even if many in the West (and beyond) no longer have an active religious faith, our common mental frameworks—including ideologies, metaphysics, morals, and concepts—have been conditioned over the centuries by Judeo-Christian traditions. In that sense, these inherited ideas of dominion over nature are still operative, playing a key role in the disconnected, predatory, extractive mode of relationship that currently dominates in many Western and/or industrialised societies [21].

As emphasised above, this mode of relationship is implicated in the unfolding climate crisis. This point brings us to the question motivating this paper: what can be done to address this crisis? In that respect, one answer is to help people develop more adaptive modes of relationship. There are many possible elements to this endeavour, including political, technological, and economic ones. However, among the most foundational are the psychological and cultural dimensions—how people think about this relationship. In the terminology of this special issue, this means helping people cultivate better wellbeing literacy with respect to their relationship with nature. As noted above, if wellbeing literacy is “the vocabulary, knowledge and skills that may be intentionally used to maintain or improve the wellbeing of oneself or others” [6], this means a literacy where ‘others’ does not only mean humans, but the environment as a whole. To that end, this paper rests on the informed conjecture—based on the ongoing lexicographic project introduced below—that such literacy can be fostered through engaging with cultures who have developed and/or maintained more adaptive relationships. More specifically, this engagement here takes the form of cross-cultural linguistic enquiry, focusing on untranslatable words.

### 1.2. Exploring Untranslatability

Historically, cross-cultural research has tended to be undervalued within psychology, which over recent decades has been heavily Western-centric, and specifically American-centric. This bias was not always the case. Danziger suggests that prior to the Second World War were various centres of knowledge and practice, as well as peripheral locations where such knowledge/practice was reproduced [22]. However, the post-war dominance of the United States meant that American psychology was exported globally, effectively becoming the sole centre, with the adjective ‘American’ soon erased as superfluous. Of course, during that time, local ethnopsychologies [23]—sometimes referred to as ‘indigenous’ psychologies [24]—were and still are operational globally. However, American psychology came to dominate, meaning that its concepts, ideologies, priorities, and methods have shaped the international scene [25]. An example of this—with significance here—is that (American) English has become the default language for the field. This bias is an issue, as recognised by decades of research on the linguistic relativity hypothesis (LRH), popularly known as the Sapir–Whorf hypothesis, following the work of Sapir [26] and Whorf [27]. Central to the LRH is the claim that language shapes how people experience and understand the world. In that respect, if the field’s ideas and theories are structured around the contours of English, its knowledge is therefore to an extent also provincial and culturally-specific [28].

However, the Western-centricity of psychology is becoming more widely recognised and moreover acknowledged as problematic. For instance, Henrich and colleagues [29] published an influential paper in Nature arguing that the bulk of the research in fields like psychology is conducted on and by people who are ‘WEIRD’ (from contexts that are Western, Educated, Industrialised, Rich, and Democratic). Yet the majority of the world do not fall into that category, which raises questions regarding the generalisability and validity of such research. In that light, there are increasing efforts across academia to promote and engage in cross-cultural scholarship. One such endeavour is my own recent initiative to create a lexicography of untranslatable words [30], on which the current paper is based.

While untranslatability is a contested phenomenon, it commonly refers to a word that lacks an exact equivalent in a given other language. The value of such words is manifold. First, they assist in understanding other cultures, offering insights into their values, traditions, philosophies, and ways of being. The theoretical context here is the aforementioned LRH, the stronger version of which is linguistic determinism, where language inextricably constitutes thought, whereas the milder relativistic version simply asserts that language shapes it. In relation to untranslatability, the stronger view suggests that only people from the culture that produced a given word can truly understand or experience the phenomenon it signifies [31]. However, the milder perspective holds that such words are accessible to people outside the culture to an extent, holding some universal relevance. This latter point highlights a second aspect of interest regarding untranslatable words: beyond being informative vis-à-vis the culture that created a given word, they enrich other lexica. Indeed, cultures ‘borrowing’ words from each other is central to language development. For instance, of the more than 600,000 lexemes in the OED (Oxford English Dictionary), the percentage of borrowed words—also known as loanwords—is estimated to be as high as 41% [32].

Of particular interest here is *why* words are borrowed. Haspelmath identifies two main reasons: core versus cultural borrowings [33]. The former is when a loanword replicates a word that already exists (i.e., with similar meaning) in the recipient language. This tends to happen for sociolinguistic reasons (e.g., cultural capital associated with using foreign words). This type of borrowing is not of concern here. However, the latter category is central. This occurs when the recipient language lacks its own word for a referent (e.g., if a new practice or idea is introduced to a culture). Thus, the loanword is adopted for pragmatic reasons: it is cognitive and socially useful, allowing speakers to articulate concepts they previously struggled to. In Lehrer’s terminology, such words fill ‘semantic gaps’, i.e., “the lack of a convenient word to express what [one] wants to speak about” [34]. It is such gaps that makes words untranslatable, indicating phenomena that have been overlooked or undervalued by one’s own culture, but which another culture has identified. Thus, a central premise of my lexicography is that such words can enrich the nomological network in psychology (and English more broadly). Such augmentation is desirable for many reasons, including as a means of redressing the Western-centricity of psychology. This goal therefore intersects with that of the present paper, namely developing an enriched lexicon—and hence wellbeing literacy—with respect to our engagement with nature. More specifically, this paper focuses on the following research question: what are the dimensions of ‘eco-connection’ (i.e., adaptive modes of relationship with the environment), as revealed by untranslatable words.

## 2. Methods

In the paper establishing the lexicography [30], I identified 216 untranslatable words pertaining to wellbeing through a ‘quasi-systematic’ review of academic and grey literature (quasi in that there was insufficient material in academic journals to permit a conventional systematic review). Readers interested in the process are encouraged to consult this original paper; suffice to say that the search protocol had several elements (including examining the first 20 websites returned when entering “untranslatable words” into Google). Once the 216 words had been identified, robust definitions were sought though several sources, including on-line dictionaries, peer-reviewed academic sources, and bilingual colleagues. The words and their definitions were then analysed using grounded theory (GT), a methodology which allows theory to emerge inductively from the data via three main coding stages (open, axial and selective). In a process of open coding, the data—words and their definitions—were examined for emergent themes, assisted by other GT processes such as memoing and initial theorising. Axial coding then involved comparing themes through constant comparison, and grouping them into categories based on conceptual similarity. Six categories were produced, paired into three meta-categories: feelings (positive and ambivalent); relationships (love and pro-sociality); and development (character and spirituality). Finally, selective coding saw the identification of a ‘core’ category of wellbeing. Although applying GT in this way might be deemed unconventional, there is considerable heterogeneity in the studies purporting to use GT, and it is sufficiently aligned with GT principles to be considered one such example.

Following this initial paper, the lexicography has since expanded to over 1200 words, partly through crowd-sourced contributions to a website created to host the project (www.drtimlomas.com/lexicography), and partly through my own follow-up enquiries through ‘conceptual snowballing’. The term snowballing derives from recruitment, where participants facilitate the involvement of additional people. This metaphor has been borrowed to reflect the way enquiries into an untranslatable word might lead one to encounter related concepts. For instance, although over 120 languages are currently represented in the lexicography, many words are taken from a select group that are especially well-studied in psychologically-oriented literature, including Chinese, French, German, Greek, Japanese, Pāli and Sanskrit. Thus, an enquiry into a word from these languages would often lead me to a text in which related words are discussed (which would then be added to the lexicography). In adding a word, the same checking procedures were followed as in the initial paper. Moreover, once words and their definitions had been added, they were accessible on the website for public inspection and feedback (with people sometimes suggesting a refined definition of a word), providing a further credibility check (which is valued in GT).

This subsequent phase of data collection cannot be regarded as systematic (not even in the ‘quasi’ sense of the original paper). Indeed, some 7000 languages exist worldwide, and it is unlikely that one research project could study them all and retrieve their relevant words. However, even if the lexicography is a work-in-progress, one may still usefully analyse its existing words and emergent themes, even if such analyses are incomplete and subject to revision. Indeed, with the addition of the new words, the thematic structure outlined in the original paper has been updated. The six categories initially identified are still present, and moreover have been enriched by the additional words, with thematic analyses published on each (positive feelings [35], ambivalent feelings [36], love [37], prosociality [38], character [39], and spirituality [40]), plus a theoretical paper [28] and monograph [41] on the lexicographic project itself. However, the additional words have also led to six new categories being identified. The meta-category of feelings now also includes sensations and cognition. The meta-category of development now also includes understanding and skills. Further, the meta-category of relationships now also includes aesthetics and eco-connection. It is of course this latter category that is the focus of the present paper.

This emergent category of eco-connection comprises over 150 words at present. For this paper, these words were analysed using the GT variation developed in my original paper [30]. The data again comprised the words and their definitions, which had been refined and checked in the ways outlined above (e.g., consulting dictionaries, peer-reviewed sources and bilingual speakers, together with website feedback). In the first stage of open coding, words and their definitions were examined for thematic content. Next, words were grouped together through constant comparison into nine thematic codes (referred to below as ‘sub-themes’), and in turn aggregated into three themes. This process could be described as somewhat intuitive since, unlike factor analysis (with its recourse to statistical techniques), choosing which thematic structure provides the ‘best fit’ for the data mainly relies on the informed judgement of the researcher. Thus, it is acknowledged that this analytic process is somewhat idiosyncratic, shaped by my personal inclinations and perspectives; other researchers may have configured and labelled the themes differently, based on their own situatedness and reading of the data. Finally, a single ‘core’ category was generated, namely eco-connection (although this category had been in mind from the start, so cannot be deemed a truly inductively-derived core category).

## 3. Results and Discussion

**Figure 1:**
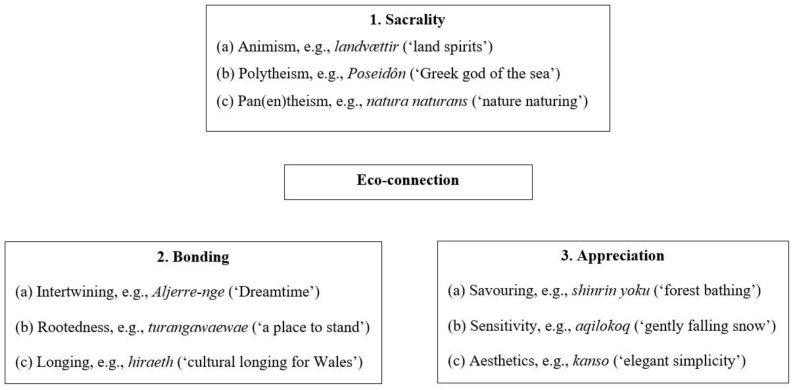
The themes and subthemes of eco-connection.

The analysis generated three emergent themes—sacrality, connection and appreciation—each of which has three subthemes, as illustrated below in Figure 1. These themes will be discussed in turn, illustrated using select examples from the lexicography (usually several per subtheme).

### 3.1. Sacrality

The first theme captures the complex and plural idea that nature is ‘sacred’ in some way. The sacred is itself a contested, evolving idea. Etymologically, it entered English around the 12th century, derived (via French) from the Latin sacrare, which encompasses meanings such as to anoint, consecrate, dedicate, immortalize, and make holy [42]. In modern scholarship, many conceptualizations rest upon the pioneering work of Durkheim [43], who contrasted it with the profane: the latter pertains to ordinary everyday life, the former to “things set apart and forbidden”. Thus, the sacred describes phenomena regarded as ‘other’ and non-ordinary. This can include divine beings, and places and objects connected to these [44]. It also encompasses phenomena simply deemed ‘numinous’ in some way [45]. That said, in Otto’s original articulation of the numinous [46]—based on the Latin numen, meaning divine power or presence—this concept was generally interpreted in theistic terms [47]. There are many untranslatable terms in this space, including proper names (e.g., of deities). Names are not usually considered examples of untranslatable words, since one would not normally ‘translate’ a name. However, they are relevant in this context if there is no equivalent in English. For instance, it is significant to the analysis that there is seemingly no English equivalent of Poseidon (Greek god of the sea). Of the various words pertaining to sacrality, these can be organized into three subthemes, based on a conventional taxonomy of forms of religion: animism, polytheism, and pantheism/panentheism.

First, cross-culturally, many of the earliest conceptions of the sacred fall under the overarching label of animism, a term coined by nineteenth-century anthropologists from the Latin anima (meaning soul, breath, or life). The label reflects the belief that all natural phenomena individually—e.g., each tree or river—possess a unique spirit or soul. Indeed, animism was probably the dominant mode of cognition among the social groups that started to coalesce approximately sixty thousand years ago (or possibly earlier) [48]. It is perhaps understandable that early societies concluded that natural phenomena possessed some sort of consciousness and soul, given that humans themselves were just starting to acquire cognisance of their own thoughts, feelings, and volition. Their world was thus ‘enchanted’, as Weber put it, suffused with agency and significance [16]. Moreover, animism is not an exclusively ancient perspective, but continues to have force today. Norse mythology, for instance—millennia in the making—constitutes a living belief system for many people of the region. To give some examples, its taxonomy is populated by a multitude of vættir (nature spirits), including landvættir (land spirits), vatnavættir (water spirits), and sjövættir (sea spirits). Indeed, Iceland celebrates four landvættir on its coat of arms (Dreki the dragon in the east, Gammur the griffin to the north, Griðungur the bull in the west, and Bergrisi the giant of the south). Such spirits are connected to specific locales, which they guard and infuse with their presence. To reiterate the point about this mythology still having resonance, Icelandic officials have been known to assess the potential impact on vættir habitats when considering urban planning [49]. Many other animist mythologies have existed and continue to exist—all with lexica of relevant untranslatable words—but the Norse example is sufficient to illustrate this first form of nature sacrality. Indeed, many such words and mythologies are not yet included in the lexicography—which, as emphasised above, is a work-in-progress—and so could not be featured here in any case.

The second class of words relating to sacrality can broadly be termed polytheistic. One should be wary though of positioning specific mythologies as exclusively one class or another. After all, Norse mythology is not wholly animistic, but also has a pantheon of gods—including Odin, Thor, and Loki—which introduces a polytheistic element. Indeed, the distinction between animism and polytheism is not always clear cut. The main difference pertains to the level of abstraction, where in polytheism the deities are somewhat removed from the phenomena they represent or rule over, existing transcendently in another realm. As with animism, there are and have been numerous polytheistic taxonomies across cultures, many featuring gods that signify or relate to nature in some way. Take Greek mythology as an example. This envisaged three separate generations of divinities, spanning aeons, as detailed in epics such as Hesiod’s Theogony (circa 700 BCE (before common era)), which outlined their genealogy [50]. First came the Prōtógonos (‘first-born’), a primeval triad of creative forces that fashioned existence. In the beginning was Kháos, the void preceding the cosmos. The cosmos then came into being through the union of Gaia and Ouranus, deities of earth and heaven. Together they created a second generation of twelve Titânes, who were subsequently overthrown by the twelve Olympian gods. The latter were contemporary with the classical era, and conceived of as exerting a powerful influence on people’s lives. Their names are legendary still, and include several pertaining to nature, including Poseidôn (god of the sea) and Dēmētēr (goddess of the harvest). Moreover, as with animism, some polytheistic deities continue to have meaning for people (even if not in the same way as for our ancestors). For instance, when Lovelock and Margulis [51] proposed their theory of ‘atmospheric homeostasis by and for the biosphere’—describing the earth’s dynamic ability to maintain viability as a living planet—they named it Gaia. Moreover, subsequently, people have been newly inspired by this theory—for instance within ecological movements—to regard the earth as sacred [52].

This latter use of Gaia shades into our third class of sacrality—reiterating the point that the distinction between the classes is not always clear-cut—namely, nature itself as sacred. There are two subtly different forms of this perspective, pantheism and panentheism. The former is arguably a form of monotheism (identifying one overarching divinity), one which views God and the cosmos as indivisible. This perspective is most closely associated with the philosopher Spinoza, who invoked the monist idea of substantia—something capable of self-subsistence—and argued that there was only one substance in the universe, namely God [53]. He then employed the Latin phrase natura naturans (‘nature naturing’) to reflect the idea that God is the dynamic process of creation itself, nature unfurling in all its glory. Subsequently, many modern thinkers have endorsed his perspective (without reference necessarily to a theistic being), where the cosmos itself is regarded as divine. For instance, asked whether he believed in God, Albert Einstein replied, “I believe in Spinoza’s God, who reveals himself in the orderly harmony of what exists, not in a God who concerns himself with the fates and actions of human beings” [54]. In addition to pantheism is also a stance known as panentheism, coined in 1828 by Karl Krause using Greek roots to mean ‘all in God’. Pantheism simply means ‘all God’, where the cosmos and the divine are one, so the divine is immanent (immersed in the cosmos) but not transcendent (it does not exist other than as the cosmos). By contrast, in panentheism, in addition to being immanent, the divine is also transcendent, thereby being both of the cosmos and outside it. An often-cited form of panentheism is the notion of Brahman, a central feature of the sacred vision expressed in the Upaniṣads (foundational works of what is now known as Hinduism, composed between 1500 and 500 BCE) [55]. That said, at the risk of complicating the picture, Hinduism can also be construed as polytheistic. However, the Upaniṣads do attempt to identify Brahman as a unifying force beneath the multiplicity and flux of life, hence it being viewed as one of the first panentheistic philosophies [56].

### 3.2. Bonding

With this second theme, human beings themselves enter the picture. People were of course implicit in the theme above, particularly in relation to pan(en)theism, in that they are part of the natural world regarded as sacred. But this second theme is more centrally about people’s relationship to nature. Cross-culturally, there are many ways of constituting and understanding this bond, and this theme includes subtle variation in that regard. Overall, though, the uniting principle is people connecting with nature in various ways—physically, experientially, cognitively, emotionally, philosophically, and spiritually [57]. This naturally stands in contrast to the modes of disconnection noted in the introduction. Of course, in one sense, all three themes are about connection with nature (or ‘eco-connection’, per the title of the paper). But this theme in particular focuses on the quality and nature of the relationship between humans and the environment, and specifically on the ways such ties can be close, intimate, and significant. In that respect, three subthemes were identified: intertwining, rootedness, and longing.

The first subtheme of ‘intertwining’ reflects the notion that humans are inextricably part of nature, in all the multidimensional ways alluded to above. Salmón calls this type of perspective a ‘kincentric ecology’, in which people “view both themselves and nature as part of an extended ecological family that shares ancestry and origins” [58]. This stance is particularly evident in cultures often referred to as ‘indigenous’ or ‘aboriginal’. Consider for instance the peoples native to Australia, who began developing culture—e.g., symbolic art—as early as 50,000 years ago [59]. During this long cultural evolution, modes of understanding began to emerge that were fundamentally characterised by this ‘intertwining’ perspective. One such example is known as Aljerre-nge, denoting the complex cultural–religious belief system of the Aranda (or Arrente) people. Other Aboriginal peoples have comparable knowledge systems known by different names, such as the Kija’s Ngarrankarni. In English, Aljerre-nge and comparable terms are sometimes rendered as ‘Dreamtime’ or ‘The Dreaming’, terms coined by Stanner in the 1950s [60]. Although the terminology has been criticised for implying unreality, among other obfuscations [61], Stanner seemingly intended to highlight the epistemological significance of dreams as means of acquiring knowledge, including receiving guidance from ancestors. But Aljerre-nge and related concepts are about more than dreaming in the literal sense of the word (i.e., cognitive activity while asleep), signifying holistic, all-encompassing ways of perceiving all life as interconnected. Stanner also coined the term ‘everywhen’ to denote this mode of understanding, encompassing past, present and future. More than a synonym for timeless, it acknowledges the ongoing relevance in these cultures of the ancestral beings and powers that shaped the world. Moreover, such traditions and epistemologies are not confined to the past, but are vibrant, complex, living ways of engaging with the world that still resonate for many indigenous people today.

A related subtheme is ‘rootedness’. If people recognise themselves as intertwined with the natural world, they are likely to identify with, and be invested in, specific regions of the earth to which they have historical connections. This notion of connection to a locale is reflected in the Māori term turangawaewae, meaning ‘a place to stand’, describing that portion of the planet one calls one’s own [62]. It is also nicely articulated by Sale in relation to the Spanish term querencia [63], which reflects that “deep sense of inner well-being that comes from knowing a particular place on the Earth; its daily and seasonal patterns, its fruits and scents, its soils and bird-songs. A place where, whenever you return to it, your soul releases an inner sigh of recognition and realisation.” Relatedly, some words celebrate the active act of ‘earthing’ or ‘grounding’ oneself in nature—investing time in the natural world, and connecting emotionally with it—like the Finnish maadoittuminen. An important aspect to this subtheme is that it often intersects with the notion of stewardship raised above, as does the first subtheme. After all, a corollary of being intertwined is people caring for this world of which they are part (assuming people have a vested interest in caring for themselves). In itself, stewardship has various aspects and forms in the words analysed here (in addition to the conceptual variation identified in the literature above). One may feel a general sense of stewardship towards the earth, as perhaps reflected in terms like Gaia. Often though, cultures speak of stewardship over specific regions to which they feel rooted. For instance, related to turangawaewae is the term mana whenua, which pertains to the mana—alluding to force, but usually also with moral or spiritual dimensions—exerted over a territory by a given people. It has thus been used in New Zealand to describe who exerts moral authority and guardianship over land. It has even had legal implications, being incorporated into legislation that deals with stewardship of natural resources [64].

The final subtheme is more melancholic, recognising that if people can bond to particular places, they can also suffer if those bonds are threatened. This issue is a specific place-based form of longing, which is a paradigmatic example of a ‘mixed’ emotion, being “a blend of the primary emotions of happiness and sadness” [65], or more evocatively, “an emotional state suffused with a melancholic sweetness” [66]. With respect to place, there is melancholy in people being separated—by choice or necessity—from the place they love. Yet, there is a redemptive possibility of being reunited; and even if reunion is not possible, the longed-for place may nevertheless always be central to a person’s identity. Such sentiments are reflected in words tied to specific places, including hiraeth (“a Welsh cultural longing for Wales” [67]); saudade (a “key Portuguese emotion” [68], and “an emotional state that pervades Brazilian culture and thought” [66]), and toska (“one of the leitmotifs of Russian literature and Russian conversation” [23]). There are also more generic forms of longing—not tied to specific places—as reflected in terms like the German Fernweh. This combines pain (Weh) and far (fern) to capture the alluring “call of faraway places” [69]. This may be anywhere one misses (including one’s homeland), but can also apply to lands unknown (thus being a counterpart to Heimweh, i.e., ‘regular’ homesickness). Fernweh is augmented by terms such as Wanderlust, which has already entered English as a loanword denoting longing for travel and adventure. As a final point, one can also express idealistic longing for lands that do not even exist—but may have previously, or may yet in the future—as reflected in terms such as the Greek Arkadíā, describing an idyllic, utopian pastoral realm, where humans live in harmony with nature.

### 3.3. Appreciation

The third theme takes us into the realm of appreciation. There were shades of this in other themes of course. In feeling rooted to a place, for instance, this would likely include elements of appreciation for it. However, appreciation is not necessarily integral to that subtheme, and could conceivably even be absent from it. Here though, appreciation comes to the fore. Once again three subthemes have been identified: savouring, sensitivity, and aesthetics.

The first subtheme relates to people actively engaging with and enjoying nature. There are many relevant words across cultures, each with their own particular nuances. Japan for instance has a tradition of shinrin yoku—‘forest bathing’—drawing on Buddhism and Shintoism, which have a rich heritage of appreciative engagement with nature [70]. It refers to the act of spending quality time in forests, and alludes to the restorative benefits of luxuriating in these spaces, literally and metaphorically. After all, a wealth of research is now emerging pointing to the impact upon wellbeing of spending time in nature, as for instance shown in Gesler’s work on ‘therapeutic landscapes’ [71]. It appears that forests may be particularly good at offering these benefits, due to factors such as air quality, quietness, and diverse stimuli. Indeed, this has long been widely recognised and moreover harnessed in Japanese clinical contexts, where shinrin yoku is offered for the treatment of physical or psychological ailments [72]. Moreover, the practice is beginning to filter into other cultures where it is also being used therapeutically [73]. Such receptivity is unsurprising given that many cultures have comparable ideas and practices. Consider for instance the Norwegian concept of friluftsliv—‘free air life’—which describes a philosophy of open-air living and being in tune with nature [74]. This notion has long been valorised by Norwegians, and Nordic nations more broadly. It is reflected in parenting and schooling practices, for example, where children regularly spend portions of the school day outside, whatever the weather; hence the saying, ‘There’s no such thing as bad weather, just bad clothing’—which is the title for a popular Nordic parenting book [75].

The second subtheme is sensitivity: being attentive and receptive to the details of the natural world. This follows from the first subtheme, in that savouring nature includes playing close attention to it, and developing a fine-grained appreciation of its nuances. An example of this lexical granularity can be found in how cultures delineate the seasons. For instance, Japanese identifies 72 distinct kō (micro-seasons) lasting five days each, such as kōgan kitaru (wild geese return), from the 8th–12th October. A related case of cultural variation—yet also commonalities—is around the traditions that have emerged with respect to seasonal change. In late October, say, are a wealth of related occasions marking the autumnal transition from summer to winter, such as Samhain, a Gaelic festival with Celtic Pagan origins. Granularity can also be found regarding features of the environment. A well-known example is the notion that Eskimo–Aleut languages possess many different words relating to snow and ice, such as aqilokoq, an Inuktitut term denoting gently falling snow. The issue is complicated, since such languages are agglutinative, creating complex words by combining morphemes. Thus, some linguists argue they do not possess greater complexity than English (for instance), since the latter can use adjectives to the same effect [76]. However, pragmatically, Eskimo culture is influenced by an environment dominated by snow and ice in ways that most English-speaking cultures are not. As such, Eskimo–Aleut languages contain many more relevant words in common usage than English. Analysing the North Sami language, for instance, Magga points out that knowledge of snow and ice is a “necessity for subsistence and survival”, and estimates over a thousand such lexemes in common usage [77].

The third subtheme brings an explicitly aesthetic dimension to the sensitivity above, focusing on the beauty and quality of the natural world. A good example is provided by Zen, a ‘branch’ of Buddhism that took root in China around the fifth century CE—when it mingled with the native Taoism—and flowered in Japan from the 12th century onwards [78]. While summarizing a tradition as rich as Zen is difficult, overall it constantly seeks to overcome the limitations of conceptual thought, and point directly into the ‘suchness’ (i.e., nature) of reality, enabling a “direct, intuitive experience” of it [79]. Central to this goal is the cultivated appreciation of certain aesthetic qualities regarded as pervading existence [80]. This includes perceiving these qualities in nature, and expressing them in art (as perfected by the nature-focused haiku of the 17th century poet Bashō, for example). An influential summary of these principles is provided by Hisamatsu in his classic Zen and the Fine Arts [81]. He identifies seven key principles: kanso (roughly defined as elegant simplicity, and absence of clutter and ornamentation); fukinsei (asymmetry or irregularity); koko (austere sublimity, or beauty in aged or weathered phenomena); shizen (spontaneous naturalness, and absence of premeditation); daisuzoku (freedom from routine); seijaku (tranquillity, stillness, and purity); and yūgen (profound grace, and obscure, ineffable depth). In Zen, these are all regarded as inherent qualities of nature, and of existence more broadly. Cultivating aesthetic sensitivity to these is therefore seen as a particularly efficacious route to the direct understanding of reality, and hence to wellbeing, and even farther to the ultimate goal of enlightenment itself [82].

## 4. Conclusions

This paper has sought to enhance our vocabulary around engagement with the natural world by exploring relevant untranslatable words. The context is the contention that the climate crisis can be traced in part to the disconnected modes of interaction with nature that have become dominant worldwide, particularly in Western and/or industrialised countries. One response to this situation is to learn from cultures and subcultures that have cultivated less destructive modes (encompassed under the rubric ‘eco-connection’), in this case by engaging with untranslatable words from their languages. As part of an ongoing project to identify such words, around 150 relevant terms were located. Three main themes were identified—each with three subthemes—as shown above in Figure 1.

This tripartite framework is a promising start in enhancing our understanding of eco-connection. However, it cannot yet be regarded as a fully-fledged theory, since that would be beyond the remit of the analysis here. For a start, the lexical search undertaken remains partial and a work-in progress, given that the lexicography currently only features around 120 languages, out of some 7000 worldwide. There are thus likely to be many relevant terms missing from the analysis and the lexicography as it stands. Moreover, some cultures and traditions have been considered in more depth than others (e.g., Zen), reflecting my interests, which drove the conceptual snowballing in certain directions. As such, the analysis is not a complete account of all the potential words that exist pertaining to eco-connection. It is rather an imperfect snapshot of the current lexicography with respect to this emergent category, one that is partial and subject to revision. Thus, further research will be needed, both to develop the lexicography more generally, and also to substantiate and refine this analysis of eco-connection specifically. To that end, several avenues of research have been identified and are starting to be pursued. For instance, I have been awarded a grant to work with specialists in machine learning to use such methods to progress the lexicography (e.g., identifying and analysing relevant words). Plans are also underway to devise a research program of in-depth interviews with speakers across the world’s languages (ideally covering at least one language per country). With such initiatives, it is possible that the analysis above will be refined and updated (e.g., new themes or subthemes relating to eco-connection may be identified). The presentation above also has other limitations too besides some cultures and languages not yet being included in the analysis. For instance, it would be possible to structure the thematic analysis in other ways. In fact, at earlier points in the analysis, other thematic solutions were identified. Consideration was initially given to an additional sub-theme of ‘immersion’ in nature, for example, but in the end, it was felt this could simply be enfolded within the subtheme of savouring. As a final point on limitations, the elucidation of the words here has been inevitably restricted by attempting an overarching comparative analysis within the constraints of a brief article. Translation is always a problematic exercise, so it will not have been possible to arrive at definitions that would satisfy all speakers of the donor language. Given the fluidity and complexity of language use, there are generally numerous ways of interpreting a given word. Thus, the descriptions here are merely one way of elucidating these terms, based on my interpretation of the source material. That said, dictionaries and scholarly sources were consulted in the aim of arriving at valid descriptions.

However, even with its limitations, the analysis above is still useful in providing a vocabulary with which to better understand and articulate this important notion of eco-connection. In turn, the development of such a vocabulary may hopefully play a role—however minor—in helping humankind develop more constructive relationships with nature. Indeed, I believe there to be an appetite for this kind of endeavour, and moreover evidence it can bear fruit, with benefits to public health. A case in point is the work of Robert Macfarlane. In his book Landmarks [83], he charts the wealth of nature-related words found in the various tongues of the British Isles (showing that we often overlook the lexical richness in our own backyard). These include, for instance, the Gaelic term èit—from the Isle of Lewis—which denotes the act of placing pieces of quartz in a stream so that they sparkle in the moonlight and attract salmon. He followed this up with an illustrated book, The Lost Words [84], with the premise that nature-related words have been disappearing from the languages of Britain, but that efforts can be made to reanimate this vocabulary. Of particular encouragement is the sheer enthusiasm of the public response. For instance, campaigns have unfolded organically to ensure schools have copies in their library, with children embracing the project [85]. An album has also been composed to complement the book, which has been received to considerable acclaim (https://www.thelostwords.org/). While just one example, it does demonstrate the potential—and indeed the public desire—for developing more adaptive relationships with nature. Moreover, it shows the value and promise of using language in service of this goal. It is hoped this paper may also contribute to this aim, and in doing so help improve our connection with the natural world upon which our wellbeing and indeed very survival depends.
